# A deep learning model for the detection of both advanced and early glaucoma using fundus photography

**DOI:** 10.1371/journal.pone.0207982

**Published:** 2018-11-27

**Authors:** Jin Mo Ahn, Sangsoo Kim, Kwang-Sung Ahn, Sung-Hoon Cho, Kwan Bok Lee, Ungsoo Samuel Kim

**Affiliations:** 1 Department of Bioinformatics and Life Science, Soongsil University, Seoul, Korea; 2 Functional Genome Institute, PDXen Biosystems Inc., Seoul, Korea; 3 Kim’s Eye Hospital, Seoul, Korea; 4 Department of Ophthalmology, Konyang University College of Medicine, Daejeon, Korea; Bascom Palmer Eye Institute, UNITED STATES

## Abstract

**Purpose:**

To build a deep learning model to diagnose glaucoma using fundus photography.

**Design:**

Cross sectional case study Subjects, Participants and Controls: A total of 1,542 photos (786 normal controls, 467 advanced glaucoma and 289 early glaucoma patients) were obtained by fundus photography.

**Method:**

The whole dataset of 1,542 images were split into 754 training, 324 validation and 464 test datasets. These datasets were used to construct simple logistic classification and convolutional neural network using Tensorflow. The same datasets were used to fine tune pre-trained GoogleNet Inception v3 model.

**Results:**

The simple logistic classification model showed a training accuracy of 82.9%, validation accuracy of 79.9% and test accuracy of 77.2%. Convolutional neural network achieved accuracy and area under the receiver operating characteristic curve (AUROC) of 92.2% and 0.98 on the training data, 88.6% and 0.95 on the validation data, and 87.9% and 0.94 on the test data. Transfer-learned GoogleNet Inception v3 model achieved accuracy and AUROC of 99.7% and 0.99 on training data, 87.7% and 0.95 on validation data, and 84.5% and 0.93 on test data.

**Conclusion:**

Both advanced and early glaucoma could be correctly detected via machine learning, using only fundus photographs. Our new model that is trained using convolutional neural network is more efficient for the diagnosis of early glaucoma than previously published models.

## Introduction

Machine learning is a system of artificial computer intelligence that provides computers with the ability to automatically learn without being programmed. In the healthcare sector, machine learning has been used to investigate skin cancer classification, to evaluate complex genetic interactions in autism, and to perform monitoring within the intensive care unit [1–3]. A recent study of diabetic retinopathy using deep machine learning revealed that machine learning exhibited high sensitivity and specificity for the detection of diabetic retinopathy [4].

Glaucoma is a progressive optic nerve disorder consisting of various optic disc changes, such as the notching of neuroretinal rims and enlarged optic disc cupping. Notably, glaucoma is one of the leading causes of blindness [5]. Thus, an effective, early investigation of optic disc changes is important in the diagnosis of glaucoma. Several reports have proven the efficacy of machine learning in the early detection of glaucoma [6–9]. However, the previous reports have utilized optical coherence tomography (OCT), red-free retinal-nerve-fiber-layer (RNFL) photography, or visual field tests. In the clinic, fundus photography is the most familiar and easiest test. Therefore, we investigated the efficacy of machine learning and deep learning for detection of both advanced and early glaucoma, using only fundus photography. Firstly, we have used logistic classification, a traditional machine learning technique, to check the performance on discriminating glaucoma patients from normal control. Secondly, we have used GoogleNet Inception v3[10], a pre-trained model, for transfer learning of our data to check the efficacy of deep learning. Finally, we have constructed our own convolutional neural network and compared the performance.

## Methods

### Data preparation

Fundus photographs of normal and glaucoma patients were collected from Kim’s Eye Hospital. Fundus photography was performed using a non-mydriatic auto fundus camera (AFC-330, Nidek, Japan). A total of 1,542 photos were obtained, including 786 photos from normal patients and 756 photos from 467 advanced and 289 early glaucoma patients. These photos had different sizes, and thus were scaled to have fixed width size of 800 pixels. In order to produce a fixed size input necessary for machine learning models, the photos were cropped at the region of optic nerve with size of 240*X*240pixels. The normal patients exhibited normal findings on red-free RNFL photography (Vx-10; Kowa Optimed, Inc., Tokyo, Japan), OCT (Cirrus HD-OCT, Carl Zeiss Meditec Inc., Dublin, CA), and visual field test (Humphrey 740 visual field analyzer, Carl Zeiss Meditec Inc., Dublin, CA). The inclusion criteria of the glaucoma patients were as follows: typical glaucomatous visual field defects, and/or bundle defects of RNFLs on HD-OCT, and/or bundle defects of RNFLs on red-free RNFL photography. Among 756 glaucoma patients, 467 cases were in advanced stage (near total cupping of the optic nerve, with or without severe visual field loss within 10° of fixation), and 289 cases were early glaucoma (glaucomatous RNFL defects in red-free RNFL photography, without visual field defects. The classification of early glaucoma and advanced glaucoma was determined by agreement of two specialists.

For the classification of glaucoma images from normal images even with the presence of early glaucoma images, the entire set of 1,542 images were split into 754 training, 324 validation, and 464 test datasets (images) (Table 1). The test dataset comprises about 30% of the whole dataset. The remaining dataset was split to about 70% training and 30% validation datasets. The study was approved by the Institutional Review Board of Kim’s Eye Hospital and was conducted in accordance with the tenets of the Declaration of Helsinki.

**Table 1:** Sample numbers for the machine learning.

	Advanced Glaucoma	Early Glaucoma	Normal	Total
Entire Data	467	289	786	1,542
Training Data	228	141	385	754
Validation Data	98	61	165	324
Test Data	141	87	236	464

### Logistic classification

**Fig 1:**
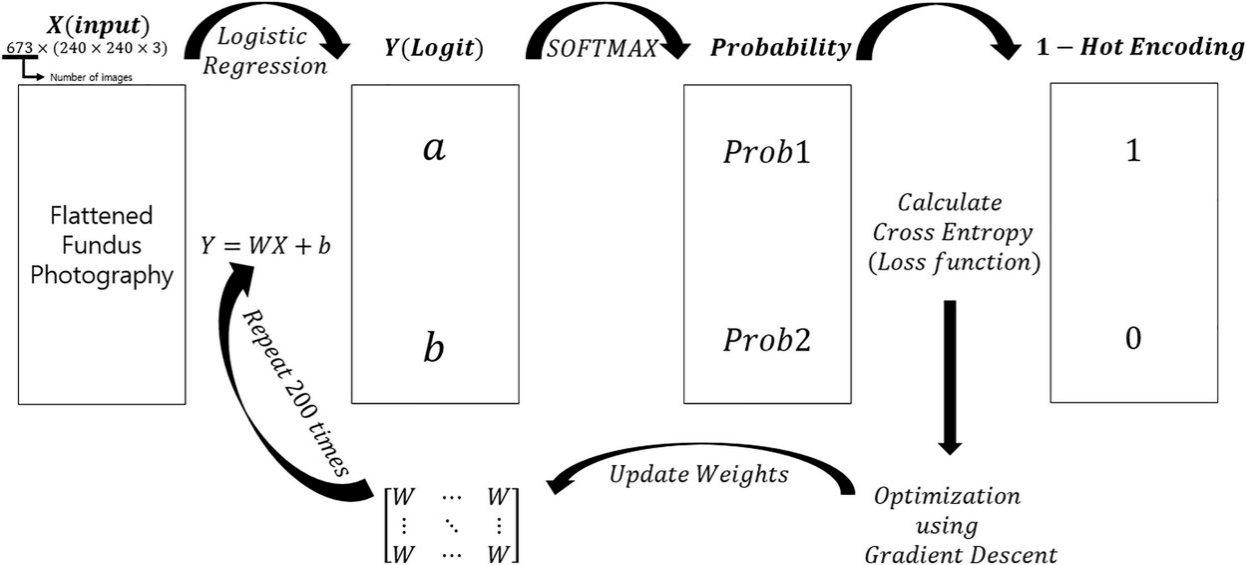
Logistic classification model architecture: A schematic view of the logistic classification model used in this study. Flattened fundus photography refers to transformation of three-dimensional array photography to a one-dimensional array in order to perform logistic regression and produce logits.

Since fundus photographs are color images, they consist of three-dimensional arrays (240×240×3). To perform logistic regression, images were flattened into a one-dimensional array of 1×(240×240×3). A single layer of weights was used to produce logits; the softmax function was applied to obtain the probability of being classified as a normal or advanced glaucoma image. These probabilities were compared to one-hot encoded labels and loss was calculated using cross entropy. A gradient descent optimizer, with a learning rate of 0.5, was used for optimization. Fig 1 shows the detailed architecture. The model was constructed using Google’s Tensorflow deep learning framework[11].

### Convolutional Neural Network (CNN)

#### Data augmentation

Since the images comprised a small dataset, we applied augmentation to each image to overcome overfitting. Each image was cropped at all four corners, as well as in the middle, to generate five images with fixed size of 224*X*224. This cropping process was repeated after flipping the image, thereby generating 10 images per photograph. Data augmentation can help overcome overfitting by showing the computer an image from various views to aid in making a decision[12].

#### Training model

**Fig 2:**
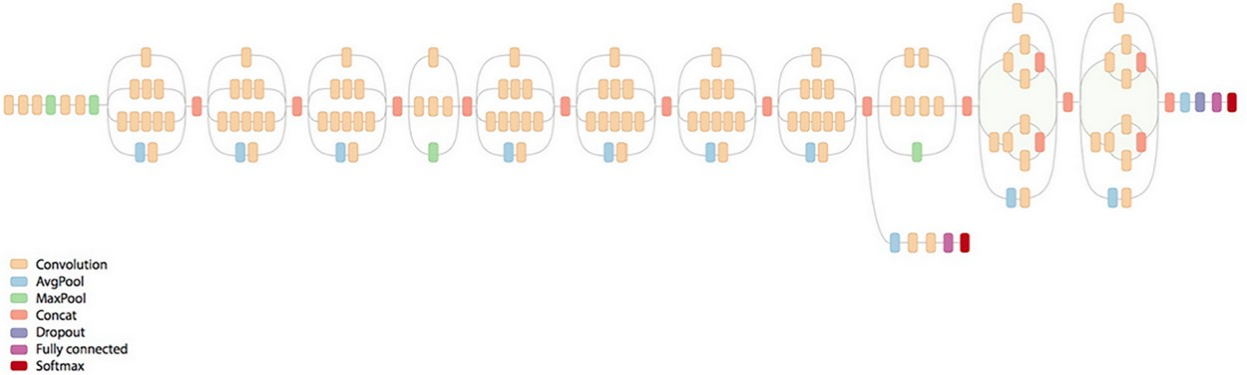
Google Inception v3 architecture: A schematic view of the Inception v3 model. Each layer consists of an inception module along with merging, and a fully connected layer at the end.

We used a GoogleNet Inception v3 pre-trained model for transfer learning, which included training our data with a predefined (trained) existing model. We modified the last classification layer of the Inception v3 model to fit our classification needs, and then fine-tuned using our data. For backpropagation, the Adam optimizer, an adaptive learning rate method, was used as an optimization function, while cross entropy was used as a loss function. Fig 2 shows the original architecture of the Inception v3 model.

**Fig 3:**
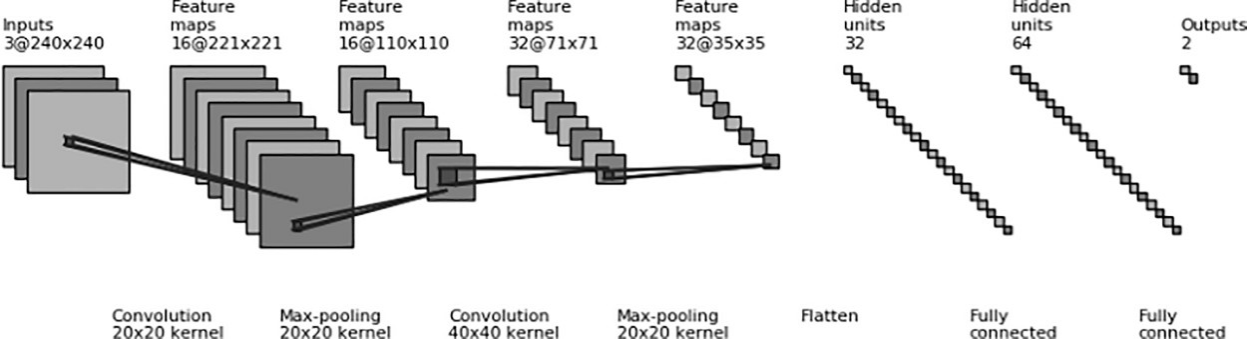
Convolutional neural network architecture: A schematic view of our convolutional neural network used in this study. It consists of three convolutional layers with max pooling applied at each layer, along with two fully connected layers.

We also constructed our own Convolutional Neural Network, using Google’s Tensorflow as backend. Two convolutional layers, with patch sizes of 2020 and 4040, were used with a stride of 1 and depths of 16 and 32. Max pooling was applied, with a patch size of 22 and a stride of 2. Two hidden layers, with 32 and 64 hidden units, were used as fully connected layers. A dropout rate of 0.5 was used in convolutional and fully connected layers to overcome overfitting; ReLu (Rectifier Linear unit) was used as an activation function. For backpropagation, cross entropy was used as a loss function and the Adagrad optimizer was used as an optimization function. All weights were initialized using the Xavier initializer[13]. Fig 3 shows the architecture of our model.

#### Evaluation

**Fig 4:**
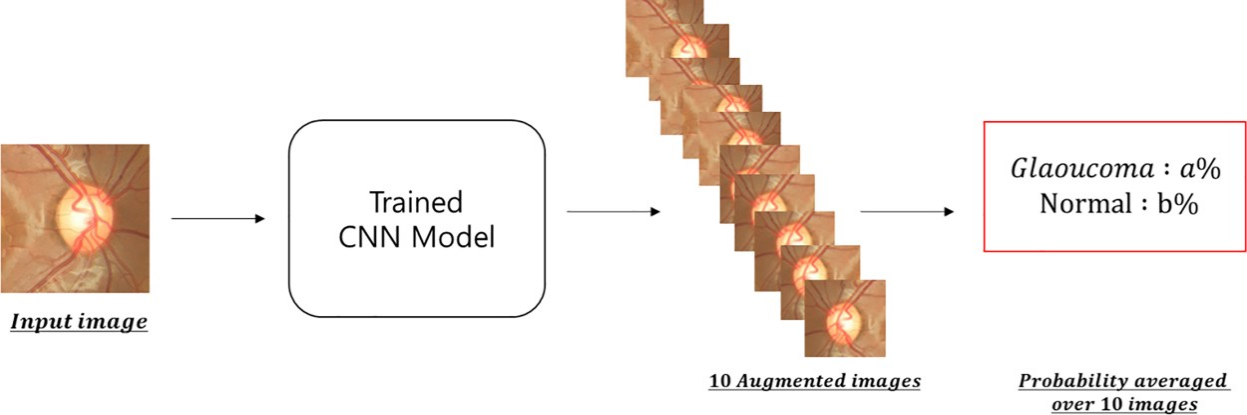
Overview of evaluation strategy: A schematic view of our convolutional neural network evaluation strategy. The probability for each augmented image is outputted by our model and averaged for the final evaluation.

Our models accept an image as input and output the probability that the image represents a photograph of a glaucoma or normal patient. Since we have used augmented data (10 images per photography), we generate 10 probabilities from a single image. By averaging this probability, we can obtain the single probability that the image represents a glaucoma or normal patient, based on each image (Fig 4). Using this strategy, we have evaluated our own model and GoogleNet Inception v3 model based on ROC (receiver operating characteristic) curve by calculating sensitivity and specificity of the models. Moreover, we measured the area under the ROC curve (AUC) as our performance indicator.

## Results

### Traditional machine learning (Logistic classification) approach

Our simple logistic classification model exhibited a training accuracy of 82.9%, a validation accuracy of 79.9% and a test accuracy of 77.2%. To check whether logistic classification model can discriminate advanced glaucoma from normal control without early glaucoma images, advanced glaucoma images were selected from entire dataset to be used to train the logistic classification model. Among 756 glaucoma images, 467 images were advanced glaucoma. 495 normal images were also selected from 786 normal images to avoid imbalanced data problem. About 30% of the 467 advanced glaucoma images and 495 normal control images were randomly split into the test dataset. This resulted in training accuracy of 99.7% and test accuracy of 98.6%. We also checked whether logistic classification model can discriminate early glaucoma from normal control without advanced glaucoma images. Among 756 glaucoma images, 289 images were early glaucoma. 289 normal images were selected from 786 normal images to avoid imbalanced data problem. About 30% of the 289 early glaucoma images and 289 normal control images were randomly split into the test dataset. This resulted in training accuracy of 83.7% and test accuracy of 73.0% (Table 2). This suggests the needs of complex algorithm such as deep learning technique to discriminate both advanced glaucoma and early glaucoma from normal control.

**Table 2:** Evaluation of the simple logistic classification model.

	Advanced and Early Glaucoma	Advanced Glaucoma only	Early Glaucoma only
Training Data	82.9%	99.7%	83.7%
Test Data	77.2%	98.6%	73%

### Deep learning (convolutional neural network) approach

**Fig 5:**
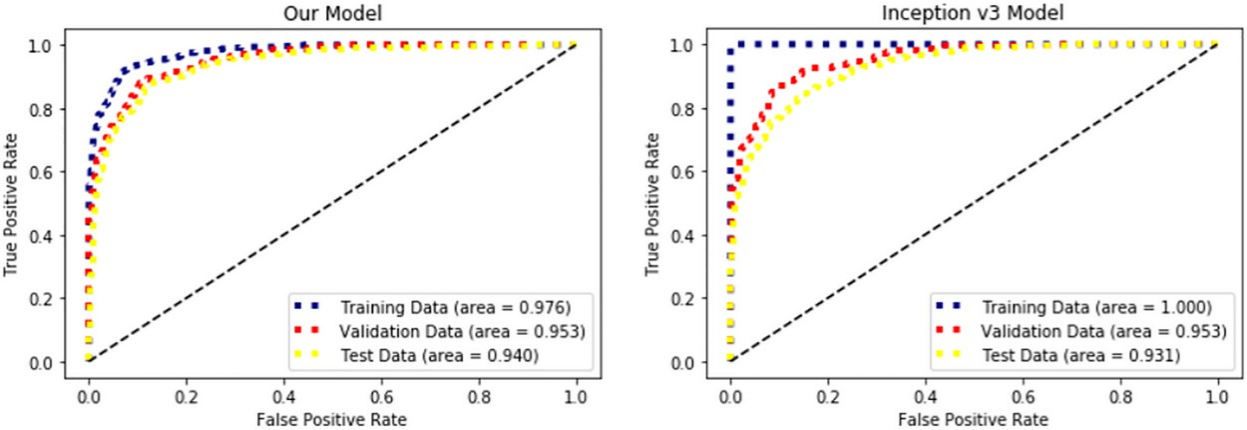
Comparative of classification performance between our Convolutional Neural Network (CNN) model and the Inception v3 model. Receiver Operating Characteristic curve for our CNN model and the transfer-learned Inception v3 model. Blue dotted line represents the training data, red dotted line represents the validation data, and yellow line represents the test data.

Table 3 shows summarized results from our own model and from the Inception v3 model. Accuracy refers to the raw accuracy of augmented data, whereas average accuracy refers to ensemble predicted accuracy (Fig 4). Inception v3 transfer learning model achieved accuracy and AUC of 99.7% and 0.99, respectively, on the training data, 87.7% and 0.95 on the validation data, and 84.5% and 0.93 on the test data. To improve test accuracy, we have developed our own convolutional neural network model. In order to build a new model, we have tuned manually various combinations of the hyper-parameters such as convolution patch size, strides, filter size, number of convolution layers, number of fully connected layers, number of hidden nodes, which optimizer to use, learning rate and so on. Our final model achieved accuracy and AUC of 92.2% and 0.98 on the training data, 88.6% and 0.95 on the validation data, and 87.9% and 0.94 on the test data. Both our own model and Inception v3 transferred model showed slightly higher ensemble accuracy than raw accuracy. The ROC curve for each model is depicted in Fig 5. The training stage was considered finished when the average loss for each epoch started to increase for the validation data. Our Convolutional Neural Network needed 29 epochs for optimization whereas Inception v3 transferred model needed 14 epochs for optimization.

**Table 3:** Comparison of our own model (convolutional neural network) and the Inception v3 model.

	Our new model			Inception v3		
	Raw accuracy	Average accuracy	AUROC	Raw accuracy	Average accuracy	AUROC
Training data	91.7%	92.2%	0.98	99.7%	99.7%	0.99
Validation data	88.6%	88.6%	0.95	87.0%	87.7%	0.95
Test Data	86.9%	87.9%	0.94	83.9%	84.5%	0.93
Number of Epochs	29			14		

## Discussion

This study demonstrates that deep learning techniques can be combined with fundus photography as an effective approach to distinguish between normal controls and glaucoma patients, even at early stages. A simple traditional machine learning approach, such as logistic classification, was sufficient for classifying advanced glaucoma patients. However, discrimination of both advanced glaucoma and early glaucoma from normal control required a complex deep learning approach, such as CNN. Using a complex deep learning model yields a vast array of parameters that may cause overfitting of the training data. Thus, we incorporated regularization techniques, such as dropout and data augmentation. Dropout randomly corrupts hidden nodes, which are passed to the succeeding layers. Since this process is random, the detailed architecture of the model changes at each iteration of training, leading to a generalized model with a sufficient number of training iterations. Data augmentation allows the machine to learn an image from a different view; using this approach, we generated 10 images per fundus photograph and averaged the results for the final evaluation. This ensemble prediction process yielded an improved model (Table 3).

Transfer learning, using the Google Inception v3 model, required less epochs for training than our CNN model. Since transfer learning requires the use of an existing trained model, all the parameters that are provided within Inception v3 were used as initial parameters[14]. Notably, these parameters have already been optimized for detecting natural images, such as edges and curves, and may require fewer epochs for optimization than a model that began from random parameters. Further, considering that the Inception v3 models are trained using extremely large numbers of images (approximately 1.28 million images), the initial convolutional patches are more generalized at detecting features; thus, these will provide a more generalized model when trained with small amounts of data. In the case of large volume data, it may take a long time to build and optimize a new model. Therefore, many studies on developing image classification model, have used transfer learning based on the state of the art Convolutional Neural Network models [1,15,16]. These models include GoogleNet Inception v3, Very Deep Convolution Network from Visual Geometry group(VGG)[17] and ResNet[18]. Recent study using large scale fundus photography used ensemble of AlexNet[19], VGG and Inception v3 transferred learned model to classify age related eye disease[20].

While transfer learning is an attractive option in building image classification model regardless of how big the data is, an alternative strategy for a small scale data would be to develop one’s own convolutional neural network model with a simpler architecture. In fact, our convolutional neural network model with far less parameters worked slightly better than the Google Inception v3 transfer learning model in terms of test accuracy (Table 3). This may be due to complexity of the Inception v3 algorithm. Since our purpose is to discriminate glaucoma and normal control, a model architecture like Inception v3, which is designed to classify 1000 categories, can be too heavy. On the other hand, our model, which was specifically tuned in terms of architecture for binary classification of glaucoma and normal control, showed improved test accuracy on a small scale. However, many trials and errors, along with a substantial amount of hyper-parameter tuning time, were required to build a new, optimized model.

Due to the complexity of neural network and many feature maps created during training time, further analysis is required to explain why a machine-trained model classified the image as a glaucoma or normal patient. Such analysis can be performed by viewing the image after each convolutional layer, along with plotting the image using a technique such as t-distributed Stochastic Neighboring Embedding (t-SNE)[21]. However, this method does not provide a score of variable importance, as in the random forest technique; thus, it may require an expert assistance.

Kim et al[22]. reported that the classification accuracy, sensitivity, specificity, and AUC for glaucoma, using machine learning, were 0.98, 0.983, 0.975, and 0.979, respectively. However, their study used multimodal imaging, including fundus photography, red-free fundus photography, visual field testing, and spectral domain OCT. While their study showed higher accuracy, sensitivity, and AUC than the present study, the latter used only fundus photography, achieving similar accuracy in cases of advanced glaucoma and only a slight difference in cases of early glaucoma.

In conclusion, deep learning using only fundus photography could be an ancillary test for the diagnosis of glaucoma. In addition, if the algorithm becomes more sophisticated, it may serve as a robust aid for detection of the early stages of glaucoma.

## References

[1] Esteva A, Kuprel B, Novoa RA, Ko J, Swetter SM, Blau HM, et al Dermatologist-level classification of skin cancer with deep neural networks. Nature. 2017;542(7639):115–118. 10.1038/nature21056 28117445 10.1038/nature21056 PMC8382232

[2] Uddin M, Tammimies K, Pellecchia G, Alipanahi B, Hu P, Wang Z, et al Brain-expressed exons under purifying selection are enriched for de novo mutations in autism spectrum disorder. Nat Genet. 2014;46(7):742–747. 10.1038/ng.2980 24859339 10.1038/ng.2980

[3] Clifton L, Clifton DA, Pimentel MA, Watkinson PJ, Tarassenko L. Gaussian processes for personalized e-health monitoring with wearable sensors. IEEE Trans Biomed Eng. 2013;60(1):193–197. 10.1109/TBME.2012.2208459 23268532 10.1109/TBME.2012.2208459

[4] Gulshan V, Peng L, Coram M, Stumpe MC, Wu D, Narayanaswamy A, et al Development and Validation of a Deep Learning Algorithm for Detection of Diabetic Retinopathy in Retinal Fundus Photographs. JAMA. 2016;316(22):2402–2410. 10.1001/jama.2016.17216 27898976 10.1001/jama.2016.17216

[5] Chua J, Baskaran M, Ong PG, Zheng Y, Wong TY, Aung T, et al Prevalence, Risk Factors, and Visual Features of Undiagnosed Glaucoma: The Singapore Epidemiology of Eye Diseases Study. JAMA Ophthalmol. 2015;133(8):938–946. 10.1001/jamaophthalmol.2015.1478 26043441 10.1001/jamaophthalmol.2015.1478

[6] Chan K, Lee TW, Sample PA, Goldbaum MH, Weinreb RN, Sejnowski TJ. Comparison of machine learning and traditional classifiers in glaucoma diagnosis. IEEE Trans Biomed Eng. 2002;49(9):963–974. 10.1109/TBME.2002.802012 12214886 10.1109/TBME.2002.802012

[7] Goldbaum MH, Sample PA, Chan K, Williams J, Lee TW, Blumenthal E, et al Comparing machine learning classifiers for diagnosing glaucoma from standard automated perimetry. Invest Ophthalmol Vis Sci. 2002;43(1):162–169. 11773027

[8] Bizios D, Heijl A, Hougaard JL, Bengtsson B. Machine learning classifiers for glaucoma diagnosis based on classification of retinal nerve fibre layer thickness parameters measured by Stratus OCT. Acta Ophthalmol. 2010;88(1):44–52. 10.1111/j.1755-3768.2009.01784.x 20064122 10.1111/j.1755-3768.2009.01784.x

[9] Barella KA, Costa VP, Gonçalves Vidotti V, Silva FR, Dias M, Gomi ES. Glaucoma Diagnostic Accuracy of Machine Learning Classifiers Using Retinal Nerve Fiber Layer and Optic Nerve Data from SD-OCT. J Ophthalmol. 2013;2013:789129 10.1155/2013/789129 24369495 10.1155/2013/789129 PMC3863536

[10] Szegedy C, Vanhoucke V, Ioffe S, Shelns J, Wojna Z. Rethinking the Inception Architecture for Computer Vision. 2016:2818–2826.

[11] Abadi M, Agarwal A, Barham P, Brevdo E, Chen Z, Citro C, et al Tensorflow: Large-scale machine learning on heterogeneous distributed systems. arXiv preprint arXiv:160304467 2016.

[12] Wong SC, Gatt A, Stamatescu V, McDonnell MD. Understanding Data Augmentation for Classification: When to Warp? 2016 Int Conf Digit Image Comput Tech Appl DICTA 2016. 2016. 10.1109/DICTA.2016.7797091

[13] Glorot X, Bengio Y. Understanding the difficulty of training deep feedforward neural networks. PMLR 2010;9:249–56.14.

[14] George D, Shen H, Huerta E. Deep Transfer Learning: A new deep learning glitch classification method for advanced LIGO. arXiv preprint arXiv:170607446 2017.

[15] York N, Overview AD. Deep Convolutional Neural Network Based Screening And Assessment Of Age-Related Macular Degeneration From Fundus Images. 2018;(Isbi):1525–1528.

[16] Zhang D. Deeply Supervised ResNet. 2017 IEEE SmartWorld, Ubiquitous Intell Comput Adv Trust Comput Scalable Comput Commun Cloud Big Data Comput Internet People Smart City Innov. 2017;(c):1–6.

[17] Simonyan K, Zisserman A. Very Deep Convolutional Networks for Large-Scale Image Recognition. 2014:1–14. 10.1016/j.infsof.2008.09.005

[18] Wu S, Zhong S, Liu Y. Deep residual learning for image steganalysis. Multimed Tools Appl. 2017:1–17. 10.1007/s11042-017-4440-4

[19] Krizhevsky A, Sutskever I, Hinton GE. ImageNet Classification with Deep Convolutional Neural Networks. Adv Neural Inf Process Syst. 2012:1–9. 10.1016/j.protcy.2014.09.007.

[20] Grassmann F, Mengelkamp J, Brandl C, Harsch S, Zimmermann ME, Linkohr B, et al A Deep Learning Algorithm for Prediction of Age-Related Eye Disease Study Severity Scale for Age-Related Macular Degeneration from Color Fundus Photography. Ophthalmology. 2018:1–11. 10.1016/j.ophtha.2018.02.037 29653860 10.1016/j.ophtha.2018.02.037

[21] Lvd Maaten, Hinton G. Visualizing data using t-SNE. Journal of machine learning research 2008;9(Nov):2579–2605.

[22] Kim SJ, Cho KJ, Oh S. Development of machine learning models for diagnosis of glaucoma. PLoS One 2017;12(5):e0177726 10.1371/journal.pone.0177726 28542342 10.1371/journal.pone.0177726 PMC5441603

