# Correction: A deep learning model for the detection of both advanced and early glaucoma using fundus photography

**DOI:** 10.1371/journal.pone.0211579

**Published:** 2019-01-25

**Authors:** Jin Mo Ahn, Sangsoo Kim, Kwang-Sung Ahn, Sung-Hoon Cho, Kwan Bok Lee, Ungsoo Samuel Kim

There are errors in the second and third sentence of the second paragraph under the subheading “Training Model” in the Methods section. The correct sentences are: Two convolutional layers, with patch sizes of 20x20 and 40x40, were used with a stride of 1 and depths of 16 and 32. Max pooling was applied, with a patch size of 2x2 and a stride of 2.

Fig 3 is incorrect. The text under both “Max-pooling” labels should read “2x2 kernel.” The authors have provided a corrected version here.

**Fig 3:**
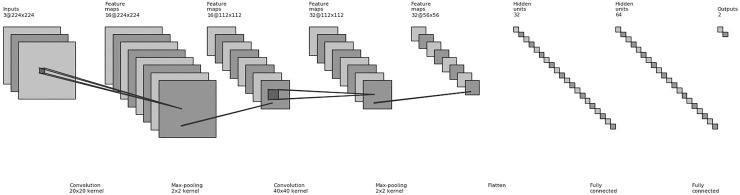
Convolutional neural network architecture: A schematic view of our convolutional neural network used in this study. It consists of three convolutional layers with max pooling applied at each layer, along with two fully connected layers.
